# P01-027 – Normal HRV in colchicine-resistant FMF patients

**DOI:** 10.1186/1546-0096-11-S1-A31

**Published:** 2013-11-08

**Authors:** U Nussinovitch, A Livneh

**Affiliations:** 1 Israel Naval Medical Institute, Haifa, Israel; 2 The Heller Institute of Medical Research, and Department of Medicine F, Sheba Medical Center, Tel Hashomer, Israel

## Introduction

The relationship between autonomic nervous system (ANS) dysfunction and Familial Mediterranean Fever (FMF) is controversial. Heart rate variability (HRV) is a powerful, simple and reliable technique for the evaluation of ANS dysfunction. Recently, we reported on normal HRV parameters, suggestive of normal ANS function, in patients with uncomplicated FMF. Also, we reported on an association between decreased HRV parameters (suggestive of ANS dysfunction), and amyloidosis of FMF, particularly at a progressive stage.

## Objectives

The aim of the current study was to evaluate whether FMF patients, who do not respond to colchicine treatment, and thereby endure persistent inflammation, have abnormal ANS function, using the HRV tool.

## Methods

Twenty-four FMF patients suffering from recurrent FMF attacks despite treatment with a maximal colchicine dose, were selected for the study. Electrocardiogram was measured under strict conditions and HRV parameters were calculated. Results were compared with age- and sex- matched unaffected controls.

## Results

No statistically significant difference was found between the groups in any determined HRV parameter: maximal RR, minimal RR and average RR intervals, standard deviation of RR interval, square root of the mean squared differences of successive RR intervals, HRV triangular index, NN50, pNN50, and power spectral analysis. Nevertheless, a statistically non-significant trend towards lower HRV parameters was observed in the colchicine non-responders group.

## Conclusion

Although a small difference in HRV parameters cannot be entirely excluded in the current study design, FMF patients, in whom colchicine did not provide adequate symptomatic relief and who did not develop amyloidosis, seem to have HRV parameters similar to those of healthy subjects, suggestive of normal ANS function.

## Disclosure of interest

None declared.

